# PET and PET/CT with ^**68**^Gallium-Labeled Somatostatin Analogues in Non GEP-NETs Tumors

**DOI:** 10.1155/2014/194123

**Published:** 2014-02-13

**Authors:** Martina Sollini, Paola Anna Erba, Alessandro Fraternali, Massimiliano Casali, Maria Liberata Di Paolo, Armando Froio, Andrea Frasoldati, Annibale Versari

**Affiliations:** ^1^Nuclear Medicine Unit, Arcispedale Santa Maria Nuova-IRCCS, Reggio Emilia, 42123 Reggio Emilia, Italy; ^2^Regional Center of Nuclear Medicine, University of Pisa, Arcispedale Santa Maria Nuova-IRCCS, Reggio Emilia, 56125 Pisa, Italy; ^3^Endocrinology Unit, Arcispedale Santa Maria Nuova-IRCCS, Reggio Emilia, 42123 Reggio Emilia, Italy

## Abstract

Somatostatin (SST) is a 28-amino-acid cyclic neuropeptide mainly secreted by neurons and endocrine cells. A major interest for SST receptors (SSTR) as target for in vivo diagnostic and therapeutic purposes was born since a series of stable synthetic SST-analouges PET became available, being the native somatostatin non feasible for clinical use due to the very low metabolic stability. The rationale for the employment of SST-analogues to image cancer is both based on the expression of SSTR by tumor and on the high affinity of these compounds for SSTR. The primary indication of SST-analogues imaging is for neuroendocrine tumors (NETs), which usually express a high density of SSTR, so they can be effectively targeted and visualized with radiolabeled SST-analogues in vivo. Particularly, SST-analogues imaging has been widely employed in gastroenteropancreatic (GEP) NETs. Nevertheless, a variety of tumors other than NETs expresses SSTR thus SST-analogues imaging can also be used in these tumors, particularly if treatment with radiolabeled therapeutic SST-analouges PET is being considered. The aim of this paper is to provide a concise overview of the role of positron emission tomography/computed tomography (PET/CT) with ^68^Ga-radiolabeled SST-analouges PET in tumors other than GEP-NETs.

## 1. Introduction

Scintigraphy with radiolabeled somatostatin (SST) analogues, first labeled with ^123^I and subsequently with ^111^In and ^99m^Tc, has proven useful in diagnosing SST-receptor- (SSTR-) positive tumors with a reported detection rate of 50–100% [1–12]. Although SSTR scintigraphy shows high efficacy for whole-body imaging, there are some limitations in organs with higher physiological uptake (e.g., liver) and in terms of detection of small lesions due to the suboptimal physical resolution of the isotopes used [13, 14]. More recently, the development of SST-analogues radiolabeled with ^68^Ga for positron emission tomography (PET) imaging such as [^68^Ga-DOTA^0^-Tyr^3^]octreotide (^68^Ga-DOTATOC, ^68^Ga-edotreotide), [^68^Ga-DOTA^0^-^1^NaI^3^]octreotide (^68^Ga-DOTANOC), and [^68^Ga-DOTA^0^-Tyr^3^]octreotate (^68^Ga-DOTATATE) has brought clear advantages compared to radiolabeled SST-analogues scintigraphy offering a higher spatial resolution and improving pharmacokinetics [15–17]. Although ^68^Ga-DOTATOC, ^68^Ga-DOTANOC, and ^68^Ga-DOTATATE can all bind to SSTR subtype 2, they have different affinity profiles for the other SSTR subtypes [18]. In particular, ^68^Ga-DOTANOC also shows a good affinity for SSTR subtypes 3 and 5, ^68^Ga-DOTATOC also binds to SSTR5 (although with lower affinity than DOTANOC), while ^68^Ga-DOTATATE has a predominant affinity for SSTR2 [19]. More recently, has been evaluated the ^68^Ga-labeled DOTA-lanreotide (DOTALAN) for which has been reported a high affinity to the SSTR subtypes 2–5 [20, 21] although other data confirmed a high affinity only for SSTR subtypes 3 and 5 [22]. The dosimetric data measured for the whole body and for specific organs using ^68^Ga-DOTATATE [23] have been published recently. Although the organ doses and effective doses for ^68^Ga-DOTATATE, and ^68^Ga-DOTATOC are similar (though ^68^Ga-DOTATOC is slightly lower), the reported dosimetry of ^68^Ga-DOTANOC is the lowest [23–25]. Importantly, the effective dose per megabecquerel for ^68^Ga-labeled SST-analogues is approximately 3–5 times lower than for ^111^In-DTPA-octreotide resulting in an additional advantage of PET tracers compared to radiolabeled SST-analogues scintigraphy [23, 26].

Finally, there was no observed toxicity, immediate or delayed, during the followup (1 year), for ^68^Ga-DOTATATE demonstrating that this radiopharmaceutical is safe and both organ-specific and effective dose exposures are acceptable [23].

The primary indication of radiolabeled SST-analogues imaging is for neuroendocrine tumors (NETs), a heterogeneous group of neoplasms that arise from endocrine cells within glands (adrenal medulla, pituitary, and parathyroid) or from endocrine islets in thyroid, pancreas, or respiratory/gastrointestinal tract, which usually express a high density of SSTR. However radiolabeled SST-analogues can also be used in the imaging of inflammatory granulomatous and autoimmune conditions as well as non NETs although they cannot be considered as the first-choice functional imaging modality in the management of these patients, except for the determination of SSTR status [27–30].  Table 1 summarizes the different SSTR subtypes expressed by each tumor considered.

The aim of this paper is to provide a concise overview of the role of positron emission tomography/computed tomography (PET/CT) with ^68^Ga-labeled SST-analogues in tumors other than GEP-NETs (Tables 2 and 3).

## 2. Sympathoadrenal System Tumors

**Figure 1:**
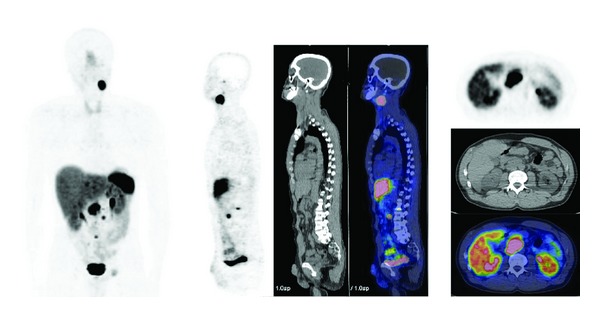
^68^Ga-DOTATOC PET/CT images (MIP, sagittal, axial) in a patient with metastatic paraganglioma.

**Figure 2:**
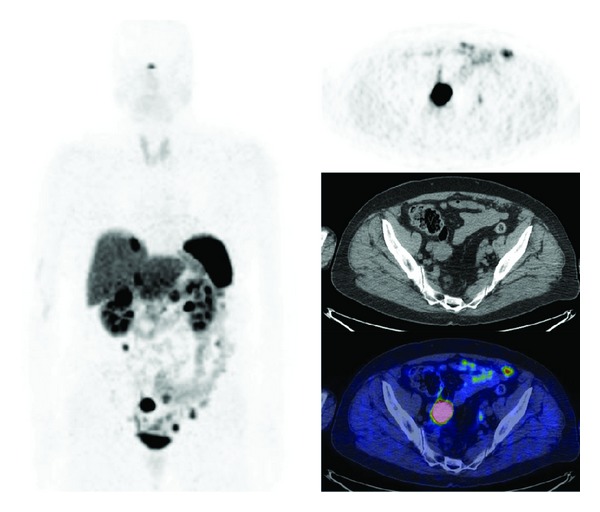
^68^Ga-DOTATOC PET/CT images (MIP, axial) in a patient affected by metastatic phaeocromochytoma.

The use of ^68^Ga-labeled SST-analogues PET and PET/CT paraganglioma (Figure 1) and phaeochromocytoma (Figure 2) remains small, consisting mainly of case reports and small series.

Fanti et al. [31] evaluated the role of ^68^Ga-DOTANOC in 14 patients with NET including 3 cases of paragangliomas. All paragangliomas were detected with ^68^Ga-DOTANOC and were strongly positive. Mittal et al. [32] retrospectively evaluated 145 patients including phaeochromocytoma (*n* = 2) and paraganglioma (*n* = 3) with ^68^Ga-DOTATATE PET/CT. PET/CT was positive in only 1 patient affected by paraganglioma. Several authors have reported the higher diagnostic performances of ^68^Ga-DOTATATE PET/CT compared to ^123^I-MIBG scintigraphy in phaeochromocytoma and paraganglioma [33–35]. Kroiss et al. [36] reported a higher sensitivity for lesion detection of ^68^Ga-DOTATOC PET/CT in metastatic phaeochromocytoma patients (*n* = 6) compared to ^123^I-MIBG scan (92% and 63%, resp.). More recently, Maurice et al. [37] reported similar results in 15 patients with phaeochromocytoma (*n* = 9) or paragangliomas (*n* = 6) evaluated with ^68^Ga-DOTATATE PET/CT and ^123^I-MIBG single photon emission computed tomography (SPECT). Utilizing ^123^I-MIBG scintigraphy as gold standard, ^68^Ga-DOTATATE had a sensitivity of 80% and a positive predictive value of 62%. The greatest discordance was in head and neck lesions, with the lesions in 4 patients being picked up by ^68^Ga-DOTATATE and missed by ^123^I-MIBG. On a per-lesion analysis, ^68^Ga-DOTATATE was superior to ^123^I-MIBG in detecting lesions in all anatomical locations (particularly bone lesions). Very recently, Sharma et al. [38] studied 26 patients with known or suspected head and neck paragangliomas by ^68^Ga-DOTANOC PET/CT and compared PET/CT findings to ^123^I-MIBG scintigraphy and CT/MRI results. ^68^Ga-DOTANOC PET/CT was positive in all patients and it was able to detect more lesions (*n* = 78) compared to ^123^I-MIBG alone or combined with CT/MRI (*n* = 30 and *n* = 53, resp.). ^68^Ga-DOTANOC PET/CT has also been compared to CT for the evaluation of bone metastases in patients with NET including patients with paraganglioma (*n* = 5), being more accurate than CT for the early identification of bone lesions [31].

Hofman et al. [39] compared ^68^Ga-DOTATATE PET/CT to ^111^In-octreotide imaging (SPECT or SPECT/CT) in a series of oncological patients including phaeochromocytoma (*n* = 4) in order to identify the management impact of incremental diagnostic information obtained from PET/CT compared with conventional staging. ^68^Ga-DOTATATE PET/CT provided additional diagnostic information in a large proportion of patients with consequent high management impact. This impact included directing patients to curative surgery by identifying the primary site and directing patients with multiple metastases to systemic therapy.

In conclusion, in case of negative ^123^I-MIBG scan in patients with a high pretest probability of phaeochromocytoma or paraganglioma, ^68^Ga-labeled SST-analogues PET or PET/CT should be considered as the next investigation. Additionally, ^68^Ga-labeled SST-analogues PET/CT should be considered in the staging of patients in whom metastatic spread, particularly to the bone, is suspected.

## 3. Lung Tumors

**Figure 3:**
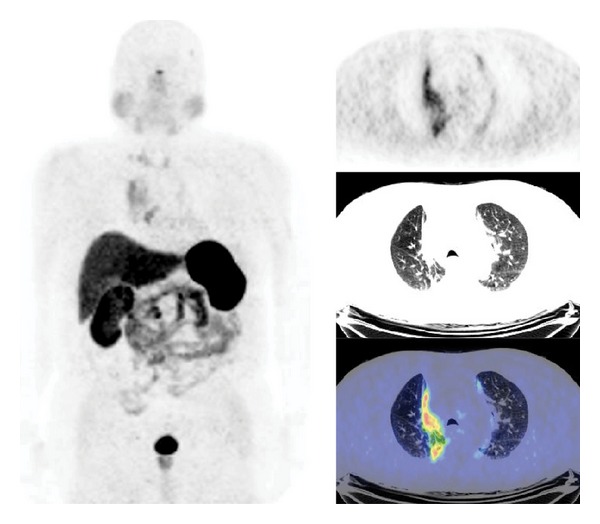
^68^Ga-DOTATATE PET/CT images (MIP, axial) in a case of metastatic atypical lung carcinoid.

**Figure 4:**
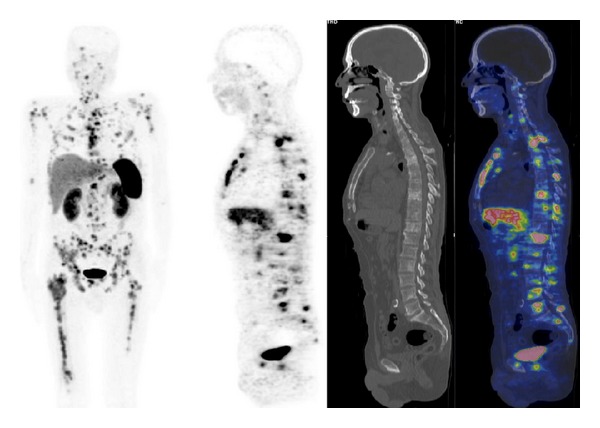
^68^Ga-DOTATOC PET/CT images (MIP, sagittal) in a patient with metastatic small cell lung carcinoma.

^68^Ga-SST-analogues PET and PET/CT have been evaluated in all types of lung tumor (Figures 3 and 4). Hofmann et al. [15] compared the diagnostic values of ^111^In-octreotide scintigraphy and ^68^Ga-DOTATOC PET to morphologic imaging in 8 patients with metastatic carcinoid tumors including 2 bronchial carcinoids. ^68^Ga-DOTATOC PET was superior to ^111^In-octreotide scintigraphy in the identification of tumor lesions (overall sensitivity of 100% versus 85%). Similarly, Koukouraki et al. [40] used ^68^Ga-DOTATOC PET to evaluate 15 cases of carcinoid tumors, including 2 cases of pulmonary carcinoids, reporting an overall sensitivity of 92%. Gabriel et al. [41] used ^68^Ga-DOTATOC PET to evaluate 84 patients with NET, including 5 patients with bronchial carcinoids, and reported results higher than those obtained with radiolabeled SST-analogues SPECT or CT. Ambrosini et al. [42] compared ^68^Ga-DOTANOC PET/CT to CT scan in 11 patients with bronchial carcinoids. There were no false-positive findings at PET/CT, and ^68^Ga-DOTANOC PET/CT detected more lesions than CT (37 versus 21). On a clinical basis, ^68^Ga-DOTANOC PET/CT provided additional information in 82% of patients changing the clinical management in 33% of cases. Kayani et al. [43] compared the performance of ^68^Ga-DOTATATE PET/CT to [^18^F]FDG-PET/CT in the detection of pulmonary NET and correlated the PET radiotracer uptake to tumor grade on histology (11 typical carcinoids, 2 atypical carcinoids, 1 large cell neuroendocrine tumor, 1 small cell neuroendocrine carcinoma, 1 non-small cell lung cancer with neuroendocrine differentiation, and 2 cases of diffuse idiopathic pulmonary neuroendocrine cell hyperplasia). All typical carcinoids showed high ^68^Ga-DOTATATE uptake (SUV_max⁡_ ≥ 8.2), but 4/11 showed negative or faint [^18^F]FDG uptake (SUV_max⁡_ = 1.7–2.9), while atypical carcinoids showed high uptake of [^18^F]FDG (SUV_max⁡_ ≥ 11.7), but 3/5 showed only faint accumulation of ^68^Ga-DOTATATE (SUV_max⁡_ = 2.2–2.8). Neither case of diffuse idiopathic pulmonary neuroendocrine cell hyperplasia showed ^68^Ga-DOTATATE or [^18^F]FDG uptake. No false-positive results were observed on ^68^Ga-DOTATATE PET/CT, while [^18^F]FDG-PET/CT was false-positive in 3 cases due to inflammation. Kumar et al. [44] compared ^68^Ga-DOTATATE and [^18^F]FDG PET/CT in 7 patients with bronchial mass detected by CT (carcinoid tumors, *n* = 3; inflammatory myofibroblastic tumor, *n* = 1; mucoepidermoid carcinoma, *n* = 1; hamartoma, *n* = 1; synovial cell sarcoma, *n* = 1). The typical carcinoids had mild [^18^F]FDG uptake and high ^68^Ga-DOTATOC uptake. Atypical carcinoid had moderate [^18^F]FDG uptake and high ^68^Ga-DOTATOC uptake. Inflammatory myofibroblastic tumor and mucoepidermoid carcinoma were positive on [^18^F]FDG-PET/CT (high and moderate uptake, resp.) and both were negative using ^68^Ga-DOTATOC PET/CT. Hamartoma showed no uptake on either [^18^F]FDG or ^68^Ga-DOTATOC PET/CT scans. Synovial cell sarcoma showed moderate [^18^F]FDG uptake and mild focal ^68^Ga-DOTATOC uptake. More recently, Jindal et al. [45] reported similar results in 20 patients with pulmonary carcinoids (13 typical and 7 atypical). In this series all the atypical carcinoids revealed higher uptake on the [^18^F]FDG-PET/CT than that in typical carcinoids while SUV_max⁡_ was significantly higher in typical carcinoids (SUV_max⁡_ = 8.8–66) compared with atypical carcinoids (SUV_max⁡_ = 1.1–18.5) on ^68^Ga-DOTATOC PET/CT. Jindal et al. [46] in a retrospective analysis of patients with primary pulmonary carcinoid (*n* = 20) who underwent ^68^Ga-DOTATOC PET/CT reported a detection rate of 95%. Putzer et al. [47] compared ^68^Ga-DOTALAN to ^68^Ga-DOTATOC PET in 53 patients with cancer including NET of the lung (*n* = 4), SCLC (*n* = 7), and bronchial carcinoid (*n* = 3). Results showed that ^68^Ga-DOTATOC has a clear advantage over ^68^Ga-DOTALAN in detection and staging of this series of NETs.

^68^Ga-SST-analogues PET/CT has also been compared to CT and bone scintigraphy for the evaluation of bone metastases in patients with lung NET being more accurate than CT and bone scintigraphy for the early identification of bone lesions [48, 49]. Finally, ^68^Ga-DOTATATE PET/CT has also been evaluated to predict progression-free survival and clinical outcome after peptide radioreceptor therapy (PRRT) in a series of patients with well-differentiated NET including 4 cases with lung NET. Results showed that patients with a decline in tumor-to-spleen SUV ratio (SUV_T/S_) after finishing the first cycle of PRRT had a significant longer time to progression than patients without favorable SUV_T/S_ changes, suggesting that this parameter has a potential role in the early prediction of outcome in patients with well-differentiated NET [50].

Dimitrakopoulou-Strauss et al. [51] compared SSTR expression assessed by ^68^Ga-DOTATOC PET to tumor viability assessed by [^18^F]FDG-PET in 9 patients with NSCLC. Moderately enhanced ^68^Ga-DOTATOC uptake was noted in 7/9 primary tumors (mean SUV_max⁡_ = 2.018 for ^68^Ga-DOTATOC and 5.683 for [^18^F]FDG) but none of the 8 metastases which were positive on [^18^F]FDG-PET showed any ^68^Ga-DOTATOC uptake. These findings suggest a loss of the SSTR expression in metastases as compared with the NSCLC primary tumors.

Recently, we evaluated the performances of PET/CT with ^68^Ga-labeled SST-analogues in 24 patients with progressive extensive SCLC, to select patients for subsequent PRRT and compared ^68^Ga-labeled SST-analogues PET/CT results to contrast-enhanced CT findings. PET/CT was positive in 83% of patients and concordant to CT findings for all the sites of disease in 37.5% of cases [52].

In conclusion, the degree of uptake and different uptake patterns on [^18^F]FDG and ^68^Ga-SST-analogues PET or PET/CT may be helpful in differentiating between typical and atypical carcinoids. ^68^Ga-SST-analogues PET/CT may be useful also to stage disease in lung cancer and to select patients for the best treatment option, including PRRT.

## 4. Brain Neuroepithelial Tissue Tumors

The overexpression of SSTR has been reported in most high grade gliomas and it may be an interesting target for PRRT. ^68^Ga-DOTATOC PET showed SSTR expression (unpublished data from Innsbruck Medical University) in the majority of patients with brain tumors (89%) including glioma (*n* = 3), medulloblastoma (*n* = 2), anaplastic astrocytoma (*n* = 1), and glioblastoma (*n* = 13) with a different degree of radiotracer uptake (faint = 37%, medium = 21%, and intense = 31%) [53]. Mittal et al. [32] retrospectively evaluated 145 patients including neuroblastoma (*n* = 8) with ^68^Ga-DOTATATE PET/CT with different purposes (initial staging, *n* = 6; disease recurrence detection and response evaluation, *n* = 1 each). In all the patients evaluated PET/CT was positive and in 5/6 cases in which ^68^Ga-DOTATATE PET/CT was performed as initial stage it was able to detect metastatic site of disease. Kroiss et al. [36] compared ^68^Ga-DOTATOC PET/CT to ^123^I-MIBG scan in a series of patients including neuroblastoma (*n* = 5) reporting the superiority of PET/CT compared to scintigraphy (sensitivity of 97% and 91%, resp.). ^68^Ga-radiolabeled SST-analogues PET/CT has been also used to select patients for PRRT (neuroblastoma, *n* = 8; glioma, *n* = 3) [54, 55] and to evaluate treatment response combined with other imaging modalities [54].

## 5. Meningioma

**Figure 5:**
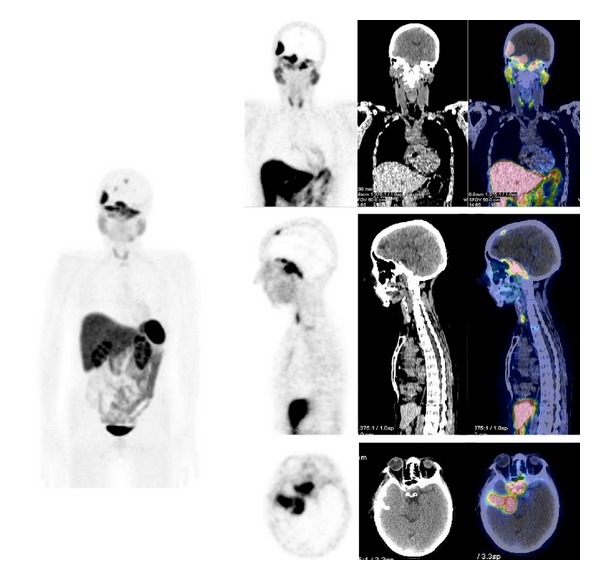
^68^Ga-DOTATATE PET/CT images (MIP, coronal, sagittal, and axial) in a patient with meningioma.

Several authors have investigated the role of ^68^Ga-labeled SST-analogues PET/CT in patients with intracranial meningioma. Virtually, all patients with meningioma present ^68^Ga-labeled SST-analogues uptake (Figure 5). Afshar-Oromieh et al. [56] compared diagnostic accuracy of ^68^Ga-DOTATOC PET/CT to brain contrast-enhanced MRI in a large series of meningioma patients before radiotherapy. In the 134 patients investigated by both modalities, 190 meningiomas were detected by ^68^Ga-DOTATOC PET/CT and 171 by contrast-enhanced MRI. With the knowledge of the PET/CT data, MRI scans were reinvestigated, leading to the detection of 4 of the 19 incidental meningiomas, resulting in an overall detection rate of 92% of the meningioma lesions that have been found by PET/CT. Milker-Zabel et al. [57] compared the planning target volume outlined on CT and contrast-enhanced MRI to the planning target volume outlined on ^68^Ga-DOTATOC PET. Patients were treated according to the planning target volume defined with CT, MRI, and PET. The planning target volume defined with CT, MRI, and PET was somewhat larger than the volume detectable in MRI/CT (median 57.2 cc and 49.6 cc, resp.). In all patients ^68^Ga-DOTATOC PET delivered additional information concerning tumor extension and the planning target volume was significantly modified based on ^68^Ga-DOTATOC PET data in 73% of the cases. Similarly, Gehler et al. [58] defined the gross tumor volume by MRI, CT, and ^68^Ga-DOTATOC PET/CT in 26 patients with meningioma. Initial gross tumor volume definition was only based on radiological data and was secondarily integrated with ^68^Ga-DOTATOC PET/CT information. ^68^Ga-DOTATOC PET/CT provided additional information concerning tumor extension in 65% of patients (especially for skull base manifestations and recurrent disease after surgery) and modified the planning target volume in more than half of patients. Nyuyki et al. [59] investigated the potential value of ^68^Ga-DOTATOC PET/CT in the definition of the gross tumor volume in 42 meningioma patients before radiotherapy. ^68^Ga-DOTATOC PET/CT findings were compared to CT and MRI. Results showed that ^68^Ga-DOTATOC PET/CT enabled delineation of SSTR-positive meningiomas and provided additional information compared to both CT and MRI regarding the planning of stereotactic radiotherapy (particularly for the detection of osseous infiltration). Additionally, in a subgroup of patients with multiple meningiomas, ^68^Ga-DOTATOC PET/CT was able to identify more lesions compared to CT or MRI (19 versus 10, resp.). Similarly, Graf et al. [60] retrospectively compared ^68^Ga-DOTATOC PET/CT to MRI and CT in the delineation of infracranial extension of skull base meningiomas in 16 patients subsequently treated with fractionated stereotactic radiotherapy. The mean infracranial volume delineable in PET was somewhat larger than the volume detectable in MRI/CT (10.1 ± 10.6 cm^3^ and 8.4 ± 7.9 cm^3^, resp.). However, authors have concluded that ^68^Ga-DOTATOC PET/CT may be useful for planning fractionated stereotactic radiation when used in addition to conventional imaging modalities often inconclusive in the skull base region. Henze et al. [61, 62] characterized meningioma with dynamic ^68^Ga-DOTATOC PET in order to evaluate kinetic parameters reporting a good correlation with MRI and CT findings and a significant difference of radiotracer uptake between meningioma and reference tissue (mean SUV = 10.5 and 1.3, resp.) suggesting a possible role of ^68^Ga-DOTATOC PET/CT in monitoring meningioma SSTR expression after radiotherapy. Recently, Hänscheid et al. [63] evaluated the predictive role of ^68^Ga-labeled SST-analogues PET to assess tumor radionuclide uptake in PRRT of meningioma. Results showed a strong correlation between SUV_max⁡_ and PRRT radionuclide tumor retention in the voxels with the highest uptake suggesting a potential role of ^68^Ga-labeled SST-analogues PET to estimate the PRRT achievable dose. Therefore ^68^Ga-labeled SST-analogues PET/CT may provide additional information in patients with uncertain or equivocal results using MRI or could help to confirm a diagnosis of meningioma based on MRI or may help to confirm MRI-based diagnosis of meningioma in cases of biopsy limitations. Finally, ^68^Ga-labeled SST-analogues PET or PET/CT may be useful to delineate the target volume for fractionated stereotactic radiotherapy.

## 6. Medullary Thyroid Cancer

**Figure 6:**
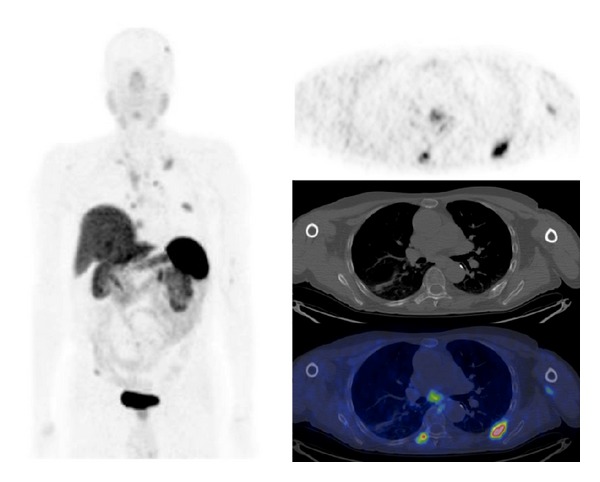
^68^Ga-DOTATATE PET/CT images (MIP, axial) in a patient affected by metastatic medullary thyroid carcinoma.

Although studies investigating larger and more homogeneous patient populations are needed to better elucidate the potential diagnostic role of new PET tracers for the assessment of recurrent medullary thyroid carcinoma (MTC), the preliminary published data seem to suggest that the diagnostic role of ^68^Ga-SST-analogues appears to be controversial (Figure 6). In fact, well-differentiated tumors show a variable and often low SSTR subtype cell expression. Of course, the evidence of a high uptake of ^68^Ga-labeled SST-analogues could be used to accurately define the tumor biology “map” and therefore may be potentially helpful in selecting the most appropriate therapeutic option. Conry et al. [64] compared the sensitivity of ^68^Ga-DOTATATE PET/CT to [^18^F]FDG-PET/CT in a series of 18 patients with recurrent MTC. Although the overall detection rate for both procedures was comparable (positive results in 72% and 77% of the cases for ^68^Ga-DOTATATE and [^18^F]FDG, resp.), on a region-based analysis [^18^F]FDG-PET identified more metastatic lesions than ^68^Ga-DOTATATE PET/CT (28 versus 23, resp.). Treglia et al. [65] retrospectively compared PET/CT with ^68^Ga-DOTATATE, [^18^F]FDG, and [^18^F]DOPA in 18 patients with residual/recurrent MTC suspected on the basis of elevated serum calcitonin levels. Results showed statistically different sensitivity values between [^18^F]DOPA and [^18^F]FDG-PET/CT (72% and 17%, resp.) and between [^18^F]DOPA and ^68^Ga-DOTATATE PET/CT (72% and 33%, resp.). Miederer et al. [66] compared a score of SSTR2 immunoistochemistry with the in vivo SUV of preoperative or prebiopsy ^68^Ga-DOTATOC PET/CT in a small series of patients including 2 patients with metastases from MTC. In these patients who were negative on immunohistochemistry PET/CT showed a moderate ^68^Ga-DOTATOC uptake (SUV_max⁡_ = 4.4 and 6.8). Koukouraki et al. [67] evaluating the pharmacokinetics of ^68^Ga-DOTATOC in series of patients with metastatic NET reported the lowest ^68^Ga-DOTATOC uptake in the patient with MTC. In another series of patients, including one case of MTC, Koukouraki et al. [40] compared ^68^Ga-DOTATOC to [^18^F]FDG PET results. In this case ^68^Ga-DOTATOC PET showed 50% of lesions evident at [^18^F]FDG-PET. Very recently, Putzer et al. [47] compared ^68^Ga-DOTALAN to ^68^Ga-DOTATOC PET in 53 patients with cancer including 8 patients with MTC. In this series of NETs ^68^Ga-DOTATOC PET showed a clear advantage over ^68^Ga-DOTALAN PET in both lesion detection and staging.

## 7. Differentiated Thyroid Carcinoma

**Figure 7:**
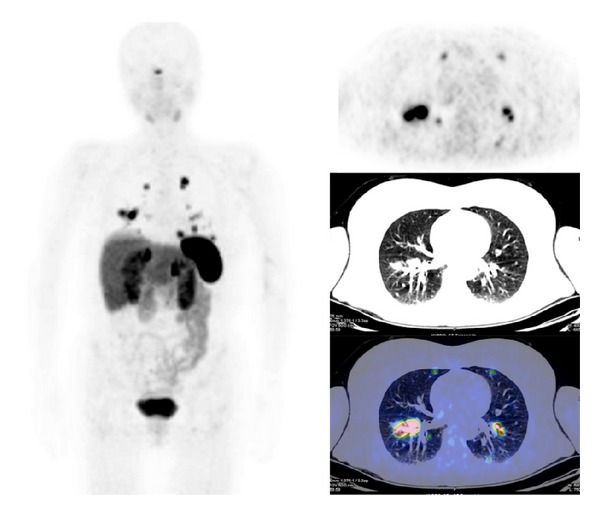
^68^Ga-DOTATATE PET/CT images (MIP, axial) in a patient with metastatic iodine-negative differentiated thyroid carcinoma.

Although papillary, follicular, and anaplastic thyroid cancers and also Hürthle-cell carcinomas do not belong to the group of traditional NET, ^68^Ga-SST-analogues PET and PET/CT may be positive in many patients (Figure 7) and could provide, especially in negative radioiodine cases, new therapeutic options. Mittal et al. [32] retrospectively evaluated 145 patients including differentiated thyroid carcinoma (DTC) patients presenting thyroglobulin-elevated negative iodine scan (*n* = 5) with ^68^Ga-DOTATATE PET/CT. In all patients evaluated, PET/CT was positive (cervical nodes, *n* = 3; remnant and cervical nodes, *n* = 1; thyroid bed soft tissue nodule, *n* = 1). Middendorp et al. [68] compared ^68^Ga-DOTATOC PET/CT to [^18^F]FDG-PET/CT in 17 patients with recurrent DTC. Both PET tracers consistently detected metastases in 12 patients. [^18^F]FDG-PET/CT has been reported more sensitive compared to ^68^Ga-DOTATOC PET/CT in the detection of radioiodine negative lesions (64% versus 31%) but not in radioiodine positive lesions (48% versus 46%). On a lesion-by-lesion basis, only 2% of lesions were visible using ^68^Ga-DOTATOC PET/CT. Gabriel et al. [69] reported the usefulness of ^68^Ga-SST analogues PET/CT to identify patients with thyroid cancer with radioiodine negative metastases (*n* = 6) suitable for PRRT. Similarly, our group used ^68^Ga-DOTATOC PET/CT to select patients with radioiodine negative metastatic DTC (*n* = 41) for PRRT [70].

## 8. Thymic Malignancies

Few data are available about the role of ^68^Ga-SST-analogues PET in thymic malignancies [40, 48, 66, 67, 71–73].

**Figure 8:**
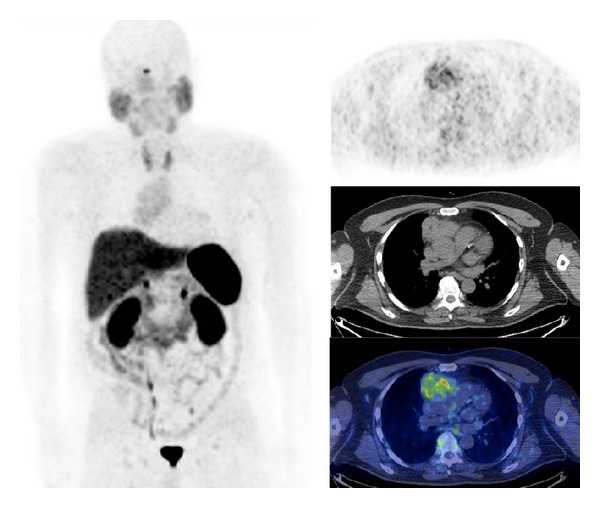
^68^Ga-DOTATATE PET/CT images (MIP, axial) in a patient with thymoma.

Miederer et al. [66] compared a score of SSTR2 immunoistochemistry with the in vivo SUV of preoperative or pre-biopsy ^68^Ga-DOTATOC PET/CT in a small series of patients including one case of thymoma. In this patient who was negative on immunohistochemistry, PET/CT showed a faint ^68^Ga-DOTATOC uptake (SUV_max⁡_ = 2.5). Dutta et al. [72] investigated 3 patients with thymic carcinoid tumors by ^68^Ga-DOTATOC PET/CT but none of these tumors showed radiotracer uptake. Koukouraki et al. [40] compared ^68^Ga-DOTATOC PET to [^18^F]FDG-PET in a series of patients including one case of carcinoid of thymus in which the disease was correctly addressed by both PET radiotracers. We reported a series of 39 patients with metastatic thymic malignancies evaluated by ^68^Ga-SST-analogues PET/CT and [^18^F]FDG-PET/CT. ^68^Ga-SST-analogues PET/CT and [^18^F]FDG-PET/CT were concordant in 43% of cases (both positive in 36% of cases and both negative in 8% of patients); in 52% of patients [^18^F]FDG-PET/CT was positive and ^68^Ga-SST-analogues PET/CT was negative while in the remaining 5% of cases ^68^Ga-SST-analogues PET/CT was positive and [^18^F]FDG-PET/CT was negative. In a per-lesion analysis, all lesions shown by contrast enhanced CT scan, which was considered the gold standard, were detected in 20% and 43% of cases using ^68^Ga-SST-analogues and [^18^F]FDG, respectively; in the remaining cases we observed at least one measurable CT lesion without either ^68^Ga-SST-analogues or [^18^F]FDG uptake. In this series of thymic neoplasms at restaging a predominant [^18^F]FDG positivity was observed compared to ^68^Ga-SST-analogues at PET/CT suggesting a relative loss of SSTR expression during thymic malignancies progression and a subsequent increasing of biological aggressiveness [73] (Figure 8).

## 9. Merkel Cell Carcinoma

Merkel cell tumors are aggressive neoplasms that often metastasize and, despite therapy, the disease-related death rate is high. Ultrastructurally and immunocytochemically, the majority of these tumors have neuroendocrine characteristics. Establishing the extent of the disease may ensure an optimal choice of treatment for these tumors; however, due to the rarity of these tumors, few cases have been evaluated by ^68^Ga-labeled SST-analogues PET/CT. Nevertheless, available data showed the usefulness of ^68^Ga-labeled SST-analogues PET/CT to stage and restage patients with Merkel cell carcinoma, and also to identify patients suitable for PRRT and to evaluate treatment response [48, 67, 74–77].

## 10. Breast Cancer

**Figure 9:**
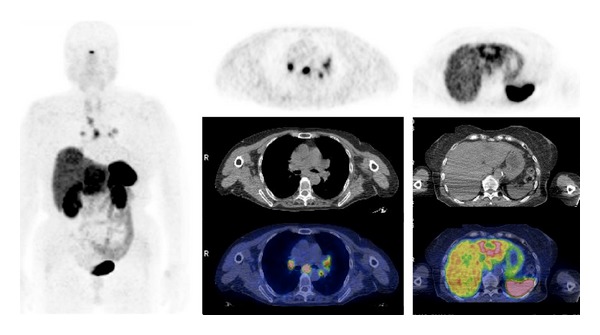
^68^Ga-DOTATOC PET/CT images (MIP, axial) in a patient with metastatic breast cancer.

In breast cancer differentiated tumors express more SSTR2 than undifferentiated ones, and estrogens positively affect SSTR2 expression; additionally, the research of new factors that could allow a more accurate prognosis of the existing disease and that could improve traditional treatment strategies remains critical [29]. However no sufficient data are available about the role of ^68^Ga-SST-analogues PET or PET/CT in this clinical setting (Figure 9). Elgeti et al. [78] retrospectively analyzed ^68^Ga-DOTATOC PET/CT performed for staging purpose in 33 women with NET. In 6/33 patients ^68^Ga-DOTATOC PET/CT revealed the presence of a breast lesion classified as suspected in 4/6 cases. In 2 cases the suspected breast lesion was diagnosed as NET metastases while in the remaining 2 cases it was diagnosed as primary breast cancer resulting in a change of therapeutic management. Primary breast cancer presented a lower ^68^Ga-DOTATOC uptake compared to concomitant abdominal NET lesions. In this small series of patients ^68^Ga-DOTATOC PET/CT not only improved NET staging but also increased the chance to detect SSTR-positive breast cancer. In the case of breast lesions, authors suggested further diagnostic characterization since the confirmation of a secondary tumor impact on therapeutic management of patients.

## 11. Colorectal Cancer

Some data suggest that SSTR2 gene expression in colorectal cancer might be related to a more favorable outcome [79]. However no sufficient data are available about the role of ^68^Ga-SST-analogues PET/CT in this clinical setting [80, 81]. Desai et al. [81] reported the usefulness of molecular imaging using different PET radiotracers in order to understand NET biology and subsequently to determine the best treatment option. In this case a different tumor pattern of [^18^F]FDG and ^68^Ga-DOTATATE uptake was shown by PET examinations within the liver, resulting in synchronous colorectal cancer and pancreatic NET liver metastases.

## 12. Melanoma

Few cases have been reported in the literature about the role of ^68^Ga-labeled SST-analogues PET/CT in melanoma patients [48, 82].

Brogsitter et al. [82] compared ^68^Ga-DOTATOC PET/CT to [^18^F]FDG-PET/CT in 18 patients with metastatic melanoma. ^68^Ga-DOTATOC PET/CT was positive in 61% of the investigated patients; however, on a lesion-by-lesion basis, only 22% of [^18^F]FDG-positive metastases were seen with ^68^Ga-DOTATOC PET/CT. Further, ^68^Ga-DOTATOC uptake was only faint (mean SUV_max⁡_ = 3.1, range 1.2–4.2) compared to [^18^F]FDG (mean SUV_max⁡_ = 28.2, range 2.3–115). The exact impact of ^68^Ga-SST-analogues PET/CT on staging and management of melanoma patient remains to be determined.

## 13. Prostate Cancer

Few cases have been reported in the literature about the role of ^68^Ga-labeled SST-analogues PET/CT in prostate cancer patients [31, 41, 48, 49, 83–85]. Luboldt et al. [84] assessed SSTR expression in 20 patients with advanced prostate cancer to potentially guide SSTR-mediated therapies. On a side-by-side analysis only 30% of bone scintigraphy-positive metastases were seen with ^68^Ga-DOTATOC PET/CT. The authors concluded by suggesting further studies with different SST-analogues with a higher affinity for SSTR1 and SSTR4 (expressed by prostate cancer), not adequately addressed with DOTATOC. The only case reported in the literature using ^68^Ga-DOTATATE showed intense radiotracer uptake in bone metastases, confirming bone scan results and suggesting a potential role of ^68^Ga-DOTATATE PET/CT to guide SSTR-mediated therapies also in this clinical setting [85].

## 14. Mesenchymal Tumors

Despite the promising results only few cases have been reported in the literature about the use of ^68^Ga-labeled SST-analogues PET/CT to evaluate tumor-induced osteomalacia (phosphaturic mesenchymal tumors) [32, 39, 86–88]. In the two larger series of patients (*n* = 6 and *n* = 8, resp.) with suspicious tumor-induced osteomalacia, PET/CT demonstrated high ^68^Ga-DOTATATE uptake and localized the tumor in 75–100% of the cases evaluated [32, 88].

In this clinical setting ^68^Ga-DOTATATE PET/CT may represent the first step functional imaging to identify the site of disease but further studies are needed to confirm these preliminary results.

## 15. Lymphoma

**Figure 10:**
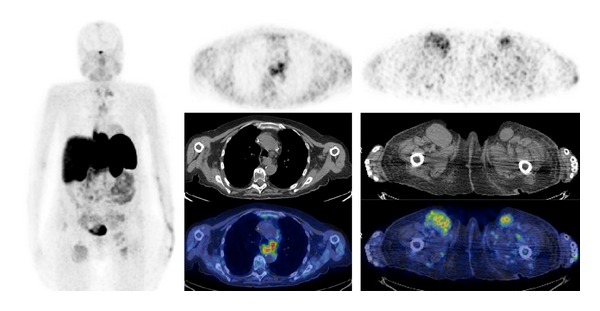
^68^Ga-DOTATATE PET/CT images (MIP, axial) in a patient with non-Hodgkin lymphoma.

The use of ^68^Ga-labeled SST-analogues PET/CT in lymphoma is limited to sporadic cases [31, 80] (Figure 10).

## 16. Conclusion and General Remarks

The use of ^68^Ga-labeled SST-analogues PET/CT in phaeochromocytoma and paraganglioma remains small, consisting mainly of case reports and small series. The diagnostic accuracy of ^68^Ga-SST-analogues PET/CT is superior to ^131^I-MIBG; thus, in the case of negative ^123^I-MIBG scan in patients with a high pretest probability of phaeochromocytoma or paraganglioma, ^68^Ga-labeled SST-analogues PET/CT should be considered. Additionally, ^68^Ga-labeled SST-analogues PET/CT should be considered in the staging of patients in whom metastatic spread, particularly to the bone, is suspected.

Although limited experience exists in NCSCL and SCLC, ^68^Ga-SST-analogues PET or PET/CT has been evaluated in all types of lung tumor. Particularly, the degree of uptake and the different uptake patterns on [^18^F]FDG and ^68^Ga-SST-analogues PET or PET/CT may be helpful to differentiate typical from atypical carcinoids. ^68^Ga-SST-analogues PET/CT may be useful also to stage lung cancer (especially for the early identification of bone lesions) and to select patients for the best treatment option, including PRRT.

Some interesting studies on radiolabeled SST-analogues PET/CT in patients with brain neuroepithelial tumors (either for staging, treatment selection, or response evaluation) are reported in the literature.

^68^Ga-labeled SST-analogues PET/CT has been widely used in patients with intracranial meningioma. ^68^Ga-labeled SST-analogues PET/CT provides additional information in patients with uncertain or equivocal results at MRI and helps to confirm a diagnosis of meningioma based on MRI or to confirm MRI-based diagnosis of meningioma in cases of biopsy limitations. Finally, ^68^Ga-labeled SST-analogues PET or PET/CT may be useful to delineate the target volume for fractionated stereotactic radiotherapy.

Although studies investigating larger and more homogeneous patient populations are needed to better elucidate the potential diagnostic role of radiolabeled SST-analogues for the assessment of recurrent MTC, the preliminary published data suggest a controversial role of ^68^Ga-SST-analogues since well-differentiated tumors show a variable and often low SSTR subtype cell expression.

^68^Ga-SST-analogues PET and PET/CT were positive in many patients with DTC providing, especially in negative radioiodine cases, new therapeutic options as PRRT. However, further studies comparing ^68^Ga-SST-analogues to radioiodine scintigraphy and [^18^F]FDG-PET/CT in DTC are needed.

Limited disappointing experience exists regarding the role of ^68^Ga-SST-analogues PET/CT in patients with thymic malignancies. In thymic neoplasms a predominant [^18^F]FDG positivity has been observed compared to ^68^Ga-SST-analogues at PET/CT suggesting a relative loss of SSTR expression during thymic malignancy progression and subsequent increasing of biological aggressiveness.

Few but significant data are available about the role of ^68^Ga-labeled SST-analogues PET/CT in Merkel cell carcinoma. ^68^Ga-labeled SST-analogues PET/CT is useful to stage and restage patients, and also to select treatment for PRRT and to assess treatment response.

Although only few cases have been reported in the literature about the use of ^68^Ga-labeled SST-analogues PET/CT in tumor-induced osteomalacia, ^68^Ga-DOTATATE PET/CT may represent the first step functional imaging to identify mesenchymal tumors; however further studies are needed to confirm the promising preliminary results.

No sufficient data are available about the role of ^68^Ga-SST-analogues PET or PET/CT in melanoma and breast, colorectal, and prostate cancers. The use of ^68^Ga-labeled SST-analogues PET/CT in lymphoma is limited to sporadic cases with unfavorable results.

In conclusion, although these preliminary experiences suggest a possible role of ^68^Ga-SST-analogues PET or PET/CT in many non GEP-NETs tumors, further studies are needed to confirm these promising results.

## Figures and Tables

**Table 1:** Somatostatin receptor subtypes expression in different tumors.

SSTR subtypes expression		References
Astrocytoma	SSTR1, SSTR2, and SSTR3 in variable percentages	[89]
Breast cancer	All of the five SSTR subtypes (predominantly SSTR2)	[90]
Colorectal cancer	Predominantly SSTR1 followed by SSTR5 and SSTR2	[91]
DTC	All of the five SSTR subtypes (predominantly SSTR2 and SSTR3)	[92]
Ependymoma	Commonly SSTR1 or SSTR5	[92]
Gastric carcinoma	Commonly SSTR2 and SSTR5, although SSTR3 is detected in several cases	[93]
GBM	Mainly SSTR3 followed by SSTR2 and SSTR1	[89]
GEP-NET	Predominantly SSTR1 and SSTR2 although SSTR5 is also often detected	[94]
GIST	All of the five SSTR subtypes in variable percentages	[95, 96]
HCC	Mainly SSTR5, although SSTR1, SSTR2, and SSTR3 are also often detected	[97]
Lymphoma	Mainly SSTR2 and SSTR3	[98]
Medulloblastoma	Mainly SSTR2	[94]
Melanoma	All of the five SSTR subtypes (predominantly SSTR1)	[99]
Meningioma	All of the five SSTR subtypes (predominantly SSTR1 and SSTR2)	[100]
Merkel cell carcinoma	Mainly SSTR2	[101]
MTC	All of the five SSTR subtypes (predominantly SSTR 2 and SSTR5)	[102, 103]
Neuroblastoma	Mainly SSTR2	[94]
NSCLC	Mainly SSTR2 and SSTR5 and, at lower level, SSTR3	[104]
Paraganglioma	Predominantly SSTR2 and SSTR1	[94]
PCa	All of the five SSTR subtypes (predominantly SSTR1)	[105, 106]
Phaeochromocytoma	Predominantly SSTR2 and SSTR1	[94]
Pituitary adenoma	Typical pattern of SSTR expression according to the secreting cells from which they originate:	[107–112]
	GH secreting: mostly SSTR2 and SSTR5, often together	
	ACTH secreting: predominantly SSTR2 together with SSTR5	
	PRL secreting: predominantly SSTR1 and SSTR5	
	TSH secreting: SSTR2 is mainly coexpressed with SSTR3 and SSTR5	
	Clinically non-functioning: SSTR3 is highly expressed, followed by SSTR2 and, at lower level, SSTR5	
Renal cell carcinoma	Mainly SSTR2	[94]
Sarcoma	Mainly SSTR2	[94]
SCLC	Mainly SSTR2	[94]

SSTR: somatostatin receptor; DTC: differentiated thyroid cancer; GBM: glioblastoma multiforme; GEP-NET: gastroenteropancreatic neuroendocrine tumor; GIST: gastrointestinal stromal tumor; HCC: hepatocellular carcinoma; MTC: medullary thyroid cancer; NSCLC: non-small cell lung cancer; PCa: prostate cancer; GH: growth hormone; ACTH: adrenocorticotropic hormone; PRL: prolactin; TSH: thyrotropin; SCLC: small cell lung cancer.

**Table 2:** Overview of the role of positron emission tomography and positron emission tomography/computed tomography with ^68^Ga-radiolabeled somatostatin analogues in tumors other than gastroenteropancreatic neuroendocrine tumors.

Reference	Tumor type	Method	Purpose	Results
Hofmann et al. 2001 [15]	Bronchial carcinoid (*n* = 2)	^ 68^Ga-DOTATOC PET	Mts detection	Overall sensitivity = 100%*

Koukouraki et al. 2006 [40]	Paraganglioma (*n* = 1); pulmonary carcinoid (*n* = 2); thymic carcinoid (*n* = 1); MTC (*n* = 1)	^ 68^Ga-DOTATOC PET (dynamic)	Evaluation of pharmacokinetics	Detection rate = 3/4 in paraganglioma = 5/5 in lung carcinoid = 3/3 in thymus carcinoid = 3/6 in MTC

Koukouraki et al. 2006 [67]	Paraganglioma (*n* = 1); pulmonary carcinoid (*n* = 2); thymic carcinoid (*n* = 2); MTC (*n* = 1); Merkel cell carcinoma (*n* = 1)	^ 68^Ga-DOTATOC PET (dynamic)	Evaluation of pharmacokinetics	Detection rate = 97%*

Gabriel et al. 2007 [41]	Paraganglioma (*n* = 3); bronchial carcinoid (*n* = 6); prostate NET (*n* = 1)	^ 68^Ga-DOTATOC PET	Staging/follow-up	Overall sensitivity = 97%*

Fanti et al. 2008 [31]	Paraganglioma (*n* = 3); prostate NET (*n* = 3); lymphoma (*n* = 1)	^ 68^Ga-DOTANOC PET/CT	Restaging/treatment planning	Positive in 4/7 cases

Ambrosini et al. 2010 [48]	Paraganglioma (*n* = 5); lung carcinoid (*n* = 44); Merkel cell carcinoma (*n* = 1); prostate NET (*n* = 2); melanoma (*n* = 1); thymic cancer (*n* = 1)	^ 68^Ga-DOTANOC PET/CT	Bone mts detection	Overall sensitivity = 100%*

Haug et al. 2010 [50]	Paraganglioma (*n* = 1); lung NET (*n* = 4)	^ 68^Ga-DOTATATE PET/CT	Outcome prediction	Decreased ^68^Ga-DOTATATE uptake in tumor after the first cycle of PRRT predicted time to progression and correlated with an improvement in clinical symptoms

Naji et al. 2011 [33]	Paraganglioma (*n* = 4); phaeochromocytoma (*n* = 7); MTC (*n* = 1)	^ 68^Ga-DOTATATE PET or PET/CT	Staging/restaging	Positive in 10/12 cases

Maurice et al. 2012 [37]	Paraganglioma (*n* = 6); phaeochromocytoma (*n* = 9)	^ 68^Ga-DOTATATE PET/CT	Diagnosis/follow-up	Overall sensitivity = 80%*

Mittal et al. 2013 [32]	Paraganglioma (*n* = 3); phaeochromocytoma (*n* = 2); neuroblastoma (*n* = 8); DTC (*n* = 5); thymic carcinoid (*n* = 1); mesenchymal tumor (*n* = 8)	^ 68^Ga-DOTATATE PET/CT	Staging/re-staging/treatment response assessment	Positive in 20/27 cases

Sharma et al. 2013 [38]	Paraganglioma (*n* = 26)	^ 68^Ga-DOTANOC PET/CT	Staging	All positive

Win et al. 2006 [34]	Phaeochromocytoma (*n* = 5)	^ 68^Ga-DOTATATE PET	Staging/re-staging	Positive in 4/5 cases

Win et al. 2007 [35]	Phaeochromocytoma (*n* = 5)	^ 68^Ga-DOTATATE PET	Staging/re-staging	Positive in 4/5 cases

Kroiss et al. 2011 [36]	Phaeochromocytoma (*n* = 6); neuroblastoma (*n* = 5)	^ 68^Ga-DOTATOC PET/CT	PRRT selection	Sensitivity = 92% for phaeochromocytoma = 97% for neuroblastoma

Hofman et al. 2012 [39]	Phaeochromocytoma (*n* = 4); mesenchymal tumor (*n* = 2)	^ 68^Ga-DOTATATE PET/CT	Staging	High/moderate management impact = 57%

Miederer et al. 2009 [66]	Lung carcinoid (*n* = 1); MTC (*n* = 2); thymoma (*n* = 1)	^ 68^Ga-DOTATOC PET/CT	Detection	Correlation between immunochemistry-SSTR2 score and SUV*

Ambrosini et al. 2009 [42]	Bronchial carcinoid (*n* = 11)	^ 68^Ga-DOTANOC PET/CT	Staging	Change in clinical management = 33%

Kayani et al. 2009 [43]	Typical carcinoid (*n* = 11); atypical carcinoid (*n* = 2); large cell neuroendocrine tumor (*n* = 1); small cell neuroendocrine carcinoma (*n* = 1); NSCLC with neuroendocrine differentiation (*n* = 1); diffuse idiopathic pulmonary neuroendocrine cell hyperplasia (*n* = 2)	^ 68^Ga-DOTATATE PET/CT	Staging/re-staging	Positive in 16/18 cases

Kumar et al. 2009 [44]	Bronchial carcinoid tumor (*n* = 3); inflammatory myofibroblastic tumor (*n* = 1); mucoepidermoid carcinoma (*n* = 1); hamartoma (*n* = 1); synovial cell sarcoma (*n* = 1)	^ 68^Ga-DOTATATE PET/CT	Bronchial mass detection	Positive in 4/7 cases

Putzer et al. 2009 [49]	Lung NET (*n* = 5); prostate NET (*n* = 1)	^ 68^Ga-DOTATOC PET	Mts detection	Overall sensitivity = 97%*

Jindal et al. 2010 [46]	Pulmonary carcinoid (*n* = 20)	^ 68^Ga-DOTATOC PET/CT	Staging	Detection rate = 95%

Jindal et al. 2011 [45]	Pulmonary carcinoid (*n* = 20)	^ 68^Ga-DOTATOC PET/CT	Staging	Detection rate = 100% for typical carcinoid = 86% for atypical carcinoid

Putzer et al. 2013 [47]	Lung NET (*n* = 4); SCLC (*n* = 7); bronchial carcinoid (*n* = 3); MTC (*n* = 8)	^ 68^Ga-DOTALAN versus ^68^Ga-DOTATOC PET	Detection/staging	Overall sensitivity = 63% for ^68^Ga-DOTALAN PET* = 78% for ^68^Ga-DOTATOC PET*

Dimitrakopoulou-Strauss et al. 2006 [51]	NSCLC (*n* = 9)	^ 68^Ga-DOTATOC PET (dynamic)	Staging/re-staging	Detection rate = 7/9 primary site = 0/8 mts

Sollini et al. 2013 [52]	SCLC (*n* = 24)	^ 68^Ga-DOTATOC/DOTATATE PET/CT	PRRT selection	Positive in 20/24 cases

Heute et al. 2010 [54]	Glioblastoma (*n* = 3)	^ 68^Ga-DOTATOC PET	PRRT selection	All positive

Waitz et al. 2011 [53]	Glioma (*n* = 33); medulloblastoma (*n* = 2); anaplastic astrocytoma (*n* = 1); glioblastoma (*n* = 13); meningioma (*n* = 22)	^ 68^Ga-DOTATOC PET	PRRT selection	Positive in 39/41 cases

Gains et al. 2011 [55]	Neuroblastoma (*n* = 8)	^ 68^Ga-DOTATATE PET/CT	PRRT selection	Positive in 6/8 cases

Henze et al. 2001 [61]	Meningioma (*n* = 3)	^ 68^Ga-DOTATOC PET (dynamic)	Evaluation of pharmacokinetics	All positive

Henze et al. 2005 [62]	Meningioma (*n* = 21)	^ 68^Ga-DOTATOC PET (dynamic)	Evaluation of pharmacokinetics before EBRT	Higher ^68^Ga-DOTATOC uptake in meningioma compared to reference tissue

Milker-Zabel et al. 2006 [57]	Meningioma (*n* = 26)	^ 68^Ga-DOTATOC PET	EBRT planning	Change in planning target volume = 73%

Gehler et al. 2009 [58]	Meningioma (*n* = 26)	^ 68^Ga-DOTATOC PET/CT	EBRT planning	Change in clinical target volume = 54%

Nyuyki et al. 2010 [59]	Meningioma (*n* = 42)	^ 68^Ga-DOTATOC PET/CT	EBRT planning	Change in gross tumor volume = 93%

Afshar-Oromieh et al. 2012 [56]	Meningioma (*n* = 134)	^ 68^Ga-DOTATOC PET/CT	Staging/re-staging	Detection rate = 100%

Graf et al. 2012 [60]	Meningioma (*n* = 16)	^ 68^Ga-DOTATOC PET/CT	EBRT planning	All positive

Hänscheid et al. 2012 [63]	Meningioma (*n* = 11)	^ 68^Ga-DOTATOC/DOTATATE PET	Prediction PRRT radionuclide retention	Significant correlations between SUV_max_ and the therapeutic uptake, SUV_max_ and the maximum voxel dose from PRRT

Conry et al. 2010 [64]	MTC (*n* = 18)	^ 68^Ga-DOTATATE PET/CT	Recurrence/mts detection	Positive in 13/18 cases

Treglia et al. 2012 [65]	MTC (*n* = 18)	^ 68^Ga-DOTATATE PET/CT	Recurrence/mts detection	Positive in 6/18 cases

Middendorp et al. 2010 [68]	DTC (*n* = 17)	^ 68^Ga-DOTATOC PET/CT	Recurrence/mts detection	Detection rate = 31% for radioiodine-negative lesions = 46% for radioiodine positive lesions

Gabriel et al. 2010 [69]	DTC (*n* = 6)	^ 68^Ga-DOTALAN/DOTATOC PET	PRRT selection	NA

Versari et al. 2013 [70]	DTC (*n* = 41)	^ 68^Ga-DOTATOC PET/CT	PRRT selection	Positive in 24/41 cases

Haug et al. 2012 [80]	DTC (*n* = 3); colorectal cancer (*n* = 1); lymphoma (*n* = 1)	^ 68^Ga-DOTATATE PET/CT	Recurrence detection	Overall sensitivity = 90%*

Schneider et al. 2012 [74]	Merkel cell carcinoma (*n* = 1)	^ 68^Ga-DOTATATE PET/CT	Staging	Positive

Schmidt et al. 2012 [75]	Merkel cell carcinoma (*n* = 2)	^ 68^Ga-DOTATATE PET/CT	PRRT selection	Both positive

Salavati et al. 2012 [76]	Merkel cell carcinoma (*n* = 1)	^ 68^Ga-DOTATOC PET/CT	PRRT selection	Positive

Epstude et al. 2013 [77]	Merkel cell carcinoma (*n* = 1)	^ 68^Ga-DOTATATE PET/CT	PRRT selection	Positive

Desai et al. 2011 [81]	Colorectal cancer (*n* = 1)	^ 68^Ga-DOTATATE PET	Detection	Positive

Elgeti et al. 2008 [78]	Breast cancer (*n* = 2)	^ 68^Ga-DOTATOC PET/CT	Detection	Both positive

Souvatzoglou et al. 2009 [83]	Prostate cancer (*n* = 1)	^ 68^Ga-DOTATOC PET/CT	Staging	Positive

Luboldt et al. 2010 [84]	Prostate cancer (*n* = 20)	^ 68^Ga-DOTATOC PET/CT	Bone mts detection	Detection rate = 30%

Alonso et al. 2011 [85]	Prostate cancer (*n* = 1)	^ 68^Ga-DOTATATE PET/CT	Mts detection	Positive

Brogsitter et al. 2013 [82]	Melanoma (*n* = 18)	^ 68^Ga-DOTATOC PET/CT	Staging/re-staging	Positive in 11/18 cases

Vasamiliette et al. 2009 [71]	Thymoma (*n* = 1)	^ 68^Ga-DOTATOC PET	PRRT selection	Positive only in primary tumor

Dutta et al. 2010 [72]	Thymic carcinoid (*n* = 3)	^ 68^Ga-DOTATOC PET/CT	Staging	All negative

Froio et al. 2013 [73]	Thymic malignancy (*n* = 39)	^ 68^Ga-DOTATOC/DOTATATE PET/CT	Staging/re-staging	Detection rate = 20%

von Falck et al. 2008 [86]	Mesenchymal tumor (*n* = 1)	^ 68^Ga-DOTANOC PET/CT	Detection	Positive

Woff et al. 2010 [87]	Mesenchymal tumor (*n* = 1)	^ 68^Ga-DOTATOC PET	Detection	Positive

Clifton-Bligh et al. 2013 [88]	Mesenchymal tumor (*n* = 6)	^ 68^Ga-DOTATATE PET/CT	Detection	All positive

PET: positron emission tomography; PET/CT: positron emission tomography/computed tomography; Mts: metastases; MTC: medullary thyroid cancer; NET: neuroendocrine tumor; PRRT: peptide radioreceptor therapy; DTC: differentiated thyroid cancer; NA: not available; NSCLC: non-small cell lung cancer; SCLC: small cell lung cancer; EBRT: external beam radiotherapy.

*Overall results (no specific results for each tumor type).

**Table 3:** Summary of the role of positron emission tomography and positron emission tomography/computed tomography with ^68^Ga-radiolabeled somatostatin analogues in tumors other than gastroenteropancreatic neuroendocrine tumors.

Tumor type	References	Publications (*n* = )	Patients (*n* = )	^ 68^Ga-somatostatin-analogues PET or PET/CT purpose			Future perspective*
				Diagnosis/staging	PRRT selection	Other	
Sympathoadrenal system tumors							
Paraganglioma	[31–33, 37, 38, 40, 41, 48, 50, 67]	10	∑53	x		x	++
Phaechromocytoma	[32–37, 39]	7	∑38	x	x	x	++
Lung tumors							
Carcinoid	[40–50, 66, 67]	13	∑140	x		x	++
NSCLC	[43, 51]	2	∑10	x		x	+/−
SCLC	[43, 47, 52]	3	∑32	x	x		+
Brain tumors							
Neuroepithelial tumor	[32, 36, 53–55]	5	∑45	x	x	x	+/−
Meningioma	[53, 55–63]	9	∑301	x		x	++
Thyroid cancers							
MTC	[33, 40, 47, 64–67]	7	∑48	x		x	++
DTC	[32, 68–70, 80]	5	∑72		x	x	++
Merkel cell carcinoma	[48, 67, 74–77]	6	∑7	x	x	x	+/−
Colorectal cancer	[80, 81]	2	∑2			x	+/−
Breast cancer	[78]	1	2	x			+/−
Prostate cancer	[31, 41, 48, 49, 83–85]	7	∑29			x	+/−
Melanoma	[48, 82]	2	∑19			x	+/−
Thymic cancer	[40, 48, 66, 67, 71–73]	8	∑51		x	x	−
Mesenchymal tumor	[32, 39, 86–88]	5	∑18	x			+
Lymphoma	[31, 80]	2	∑2			x	−

PET: positron emission tomography; PET/CT: positron emission tomography/computed tomography; PRRT: peptide radioreceptor therapy; NSCLC: non-small cell lung cancer; SCLC: small cell lung cancer; MTC: medullary thyroid cancer; DTC: differentiated thyroid cancer. *Based on literature data we classified the use of radiolabeled somatostatin-analogues PET or PET/CT as ++: suitable; +: promising; +/−: undetermined; and −: not indicated.

## References

[1] Reubi JC (2003). Peptide receptors as molecular targets for cancer diagnosis and therapy. *Endocrine Reviews*.

[2] Reubi JC, Waser B (2003). Concomitant expression of several peptide receptors in neuroendocrine tumours: molecular basis for in vivo multireceptor tumour targeting. *European Journal of Nuclear Medicine and Molecular Imaging*.

[3] Bombardieri E, Maccauro M, De Deckere E, Savelli G, Chiti A (2001). Nuclear medicine imaging of neuroendocrine tumours. *Annals of Oncology*.

[4] Olsen JO, Pozderac RV, Hinkle G (1995). Somatostatin receptor imaging of neuroendocrine tumors with indium-111 pentetreotide (OctreoScan). *Seminars in Nuclear Medicine*.

[5] Briganti V, Sestini R, Orlando C (1997). Imaging of somatostatin receptors by indium-111-pentetreotide correlates with quantitative determination of somatostatin receptor type 2 gene expression in neuroblastoma tumors. *Clinical Cancer Research*.

[6] Chiti A, Briganti V, Fanti S, Monetti N, Masi R, Bombardieri E (2000). Results and potential of somatostatin receptor imaging in gastroenteropancreatic tract tumours. *Quarterly Journal of Nuclear Medicine*.

[7] Chiti A, Fanti S, Savelli G (1998). Comparison of somatostatin receptor imaging, computed tomography and ultrasound in the clinical management of neuroendocrine gastro-entero-pancreatic tumours. *European Journal of Nuclear Medicine*.

[8] Krenning EP, Kwekkeboom DJ, Bakker WH (1993). Somatostatin receptor scintigraphy with [111In-DTPA-D-Phe1]- and [123I-Tyr3]-octreotide: the Rotterdam experience with more than 1000 patients. *European Journal of Nuclear Medicine*.

[9] Seregni E, Chiti A, Bombardieri E (1998). Radionuclide imaging of neuroendocrine tumours: biological basis and diagnostic results. *European Journal of Nuclear Medicine*.

[10] Jamar F, Fiasse R, Leners N, Pauwels S (1995). Somatostatin receptor imaging with indium-111-pentetreotide in gastroenteropancreatic neuroendocrine tumors: safety, efficacy and impact on patient management. *Journal of Nuclear Medicine*.

[11] Lebtahi R, Cadiot G, Sarda L (1997). Clinical impact of somatostatin receptor scintigraphy in the management of patients with neuroendocrine gastroenteropancreatic tumors. *Journal of Nuclear Medicine*.

[12] Bangard M, Béhé M, Guhlke S (2000). Detection of somatostatin receptor-positive tumours using the new 99mC-tricine-HYNIC-D-Phe1-Tyr3-octreotide: first results in patients and comparison with 111In-DTPA-D-Phe1-octreotide. *European Journal of Nuclear Medicine*.

[13] Kowalski J, Henze M, Schuhmacher J, Mäcke HR, Hofmann M, Haberkorn U (2003). Evaluation of positron emission tomography imaging using [^68^Ga]-DOTA-D Phe1-Tyr3- octreotidein comparison to [111In]-DTPAOC SPECT. First results in patients with neuroendocrine tumors. *Molecular Imaging and Biology*.

[14] Buchmann I, Henze M, Engelbrecht S (2007). Comparison of ^68^Ga-DOTATOC PET and 111In-DTPAOC (Octreoscan) SPECT in patients with neuroendocrine tumours. *European Journal of Nuclear Medicine and Molecular Imaging*.

[15] Hofmann M, Maecke H, Börner AR (2001). Biokinetics and imaging with the somatostatin receptor PET radioligand68Ga-DOTATOC: preliminary data. *European Journal of Nuclear Medicine*.

[16] Wild D, Schmitt JS, Ginj M (2003). DOTA-NOC, a high-affinity ligand of somatostatin receptor subtypes 2, 3 and 5 for labelling with various radiometals. *European Journal of Nuclear Medicine and Molecular Imaging*.

[17] Ambrosini V, Campana D, Bodei L (2010). ^68^Ga-DOTANOC PET/CT clinical impact in patients with neuroendocrine tumors. *Journal of Nuclear Medicine*.

[18] Antunes P, Ginj M, Zhang H (2007). Are radiogallium-labelled DOTA-conjugated somatostatin analogues superior to those labelled with other radiometals?. *European Journal of Nuclear Medicine and Molecular Imaging*.

[19] Ambrosini V, Campana D, Tomassetti P, Grassetto G, Rubello D, Fanti S (2011). PET/CT with 68Gallium-DOTA-peptides in NET: an overview. *European Journal of Radiology*.

[20] Smith-Jones PM, Bischof C, Leimer M (1999). DOTA-lanreotide: a novel somatostatin analog for tumor diagnosis and therapy. *Endocrinology*.

[21] Virgolini I, Szilvasi I, Kurtaran A (1998). Indium-111-DOTA-lanreotide: biodistribution, safety and radiation absorbed dose in tumor patients. *Journal of Nuclear Medicine*.

[22] Reubi JC, Schär J-C, Waser B (2000). Affinity profiles for human somatostatin receptor subtypes SST1-SST5 of somatostatin radiotracers selected for scintigraphic and radiotherapeutic use. *European Journal of Nuclear Medicine*.

[23] Walker RC, Smith GT, Liu E, Moore B, Clanton J, Stabin M (2013). Measured human dosimetry of ^68^Ga-DOTATATE. *Journal of Nuclear Medicine*.

[24] Hartmann H, Zöphel K, Freudenberg R (2009). Radiation exposure of patients during ^68^Ga-DOTATOC PET/CT examinations. *NuklearMedizin*.

[25] Pettinato C, Sarnelli A, Di Donna M (2008). ^68^Ga-DOTANOC: biodistribution and dosimetry in patients affected by neuroendocrine tumors. *European Journal of Nuclear Medicine and Molecular Imaging*.

[26] Krenning EP, Bakker WH, Kooij PPM (1992). Somatostatin receptor scintigraphy with indium-111-DTPA-D-Phe-1-octreotide in man: metabolism, dosimetry and comparison with iodine-123-Tyr-3-octreotide. *Journal of Nuclear Medicine*.

[27] Warner RRP (2005). Enteroendocrine tumors other than carcinoid: a review of clinically significant advances. *Gastroenterology*.

[28] Cascini GL, Cuccurullo V, Mansi L (2010). The non tumour uptake of 111In-octreotide creates new clinical indications in benign diseases, but also in oncology. *Quarterly Journal of Nuclear Medicine and Molecular Imaging*.

[29] Smith MC, Maggi M, Orlando C (2004). Somatostatin receptors in non-endocrine tumours. *Digestive and Liver Disease*.

[30] Virgolini I, Ambrosini V, Bomanji JB (2010). Procedure guidelines for PET/CT tumour imaging with ^68^Ga-DOTA-conjugated peptides: ^68^Ga-DOTA-TOC, ^68^Ga-DOTA-NOC, ^68^Ga-DOTA-TATE. *European Journal of Nuclear Medicine and Molecular Imaging*.

[31] Fanti S, Ambrosini V, Tomassetti P (2008). Evaluation of unusual neuroendocrine tumours by means of ^68^Ga-DOTA-NOC PET. *Biomedicine and Pharmacotherapy*.

[32] Mittal BR, Agrawal K, Shukla J (2013). Ga-68 DOTATATE PET/CT in neuroendocrine tumors: initial experience. *Journal of Postgraduate Medicine Education and Research*.

[33] Naji M, Zhao C, Welsh SJ (2011). ^68^Ga-DOTA-TATE PET vs.123I-MIBG in identifying malignant neural crest tumours. *Molecular Imaging and Biology*.

[34] Win Z, Rahman L, Towey D, Al-Nahhas A (2006). ^68^Ga-DOTATATE PET imaging in neuroectodermal tumours. *European Journal of Nuclear Medicine and Molecular Imaging*.

[35] Win Z, Al-Nahhas A, Towey D (2007). ^68^Ga-DOTATATE PET in neuroectodermal tumours: first experience. *Nuclear Medicine Communications*.

[36] Kroiss A, Putzer D, Uprimny C (2011). Functional imaging in phaeochromocytoma and neuroblastoma with ^68^Ga-DOTA-Tyr3-octreotide positron emission tomography and 123I-metaiodobenzylguanidine. *European Journal of Nuclear Medicine and Molecular Imaging*.

[37] Maurice JB, Troke R, Win Z (2012). A comparison of the performance of ^68^Ga-DOTATATE PET/CT and ^123^I-MIBG SPECT in the diagnosis and follow-up of phaeochromocytoma and paraganglioma. *European Journal of Nuclear Medicine and Molecular Imaging*.

[38] Sharma P, Thakar A, Suman KCS (2013). ^68^Ga-DOTANOC PET/CT for baseline evaluation of patients with head and neck paraganglioma. *Journal of Nuclear Medicine*.

[39] Hofman MS, Kong G, Neels OC, Eu P, Hong E, Hicks RJ (2012). High management impact of Ga-68 DOTATATE (GaTate) PET/CT for imaging neuroendocrine and other somatostatin expressing tumours. *Journal of Medical Imaging and Radiation Oncology*.

[40] Koukouraki S, Strauss LG, Georgoulias V, Eisenhut M, Haberkorn U, Dimitrakopoulou-Strauss A (2006). Comparison of the pharmacokinetics of ^68^Ga-DOTATOC and [18F]FDG in patients with metastatic neuroendocrine tumours scheduled for 90Y-DOTATOC therapy. *European Journal of Nuclear Medicine and Molecular Imaging*.

[41] Gabriel M, Decristoforo C, Kendler D (2007). ^68^Ga-DOTA-Tyr3-octreotide PET in neuroendocrine tumors: comparison with somatostatin receptor scintigraphy and CT. *Journal of Nuclear Medicine*.

[42] Ambrosini V, Castellucci P, Rubello D (2009). ^68^Ga-DOTA-NOC: a new PET tracer for evaluating patients with bronchial carcinoid. *Nuclear Medicine Communications*.

[43] Kayani I, Conry BG, Groves AM (2009). A comparison of ^68^Ga-DOTATATE and ^18^F-FDG PET/CT in pulmonary neuroendocrine tumors. *Journal of Nuclear Medicine*.

[44] Kumar A, Jindal T, Dutta R, Kumar R (2009). Functional imaging in differentiating bronchial masses: an initial experience with a combination of ^18^F-FDG PET-CT scan and ^68^Ga DOTA-TOC PET-CT scan. *Annals of Nuclear Medicine*.

[45] Jindal T, Kumar A, Venkitaraman B (2011). Evaluation of the role of [^18^F]FDG-PET/CT and [^68^Ga]DOTATOC-PET/CT in differentiating typical and atypical pulmonary carcinoids. *Cancer Imaging*.

[46] Jindal T, Kumar A, Venkitaraman B, Dutta R, Kumar R (2010). Role of ^68^Ga-DOTATOC PET/CT in the evaluation of primary pulmonary carcinoids. *Korean Journal of Internal Medicine*.

[47] Putzer D, Kroiss A, Waitz D (2013). Somatostatin receptor PET in neuroendocrine tumours: ^68^Ga-DOTA0, Tyr3-octreotide versus ^68^Ga-DOTA0-lanreotide. *European Journal of Nuclear Medicine and Molecular Imaging*.

[48] Ambrosini V, Nanni C, Zompatori M (2010). ^68^Ga-DOTA-NOC PET/CT in comparison with CT for the detection of bone metastasis in patients with neuroendocrine tumours. *European Journal of Nuclear Medicine and Molecular Imaging*.

[49] Putzer D, Gabriel M, Henninger B (2009). Bone metastases in patients with neuroendocrine tumor: ^68^Ga- DOTA-Tyr3-octreotide PET in comparison to CT and bone scintigraphy. *Journal of Nuclear Medicine*.

[50] Haug AR, Auernhammer CJ, Wängler B (2010). ^68^Ga-DOTATATE PET/CT for the early prediction of response to somatostatin receptor-mediated radionuclide therapy in patients with well-differentiated neuroendocrine tumors. *Journal of Nuclear Medicine*.

[51] Dimitrakopoulou-Strauss A, Georgoulias V, Eisenhut M (2006). Quantitative assessment of SSTR2 expression in patients with non-small cell lung cancer using ^68^Ga-DOTATOC PET and comparison with 18F-FDG PET. *European Journal of Nuclear Medicine and Molecular Imaging*.

[52] Sollini M, Farioli D, Froio A (2013). Brief report on the use of radiolabeled somatostatin analogs for the diagnosis and treatment of metastatic small-cell lung cancer patients. *Journal of Thoracic Oncology*.

[53] Waitz D, Putzer D, Kostron H, Virgolini IJ (2011). Treatment of high-grade glioma with radiolabeled peptides. *Methods*.

[54] Heute D, Kostron H, Von Guggenberg E (2010). Response of recurrent high-grade glioma to treatment with 90Y-DOTATOC. *Journal of Nuclear Medicine*.

[55] Gains JE, Bomanji JB, Fersht NL (2011). ^177^Lu-DOTATATE molecular radiotherapy for childhood neuroblastoma. *Journal of Nuclear Medicine*.

[56] Afshar-Oromieh A, Giesel FL, Linhart HG (2012). Detection of cranial meningiomas: comparison of Ga-DOTATOC PET/CT and contrast-enhanced MRI. *European Journal of Nuclear Medicine and Molecular Imaging*.

[57] Milker-Zabel S, Zabel-du Bois A, Henze M (2006). Improved target volume definition for fractionated stereotactic radiotherapy in patients with intracranial meningiomas by correlation of CT, MRI, and [^68^Ga]-DOTATOC-PET. *International Journal of Radiation Oncology Biology Physics*.

[58] Gehler B, Paulsen F, Öksüz MT (2009). [^68^Ga]-DOTATOC-PET/CT for meningioma IMRT treatment planning. *Radiation Oncology*.

[59] Nyuyki F, Plotkin M, Graf R (2010). Potential impact of ^68^Ga-DOTATOC PET/CT on stereotactic radiotherapy planning of meningiomas. *European Journal of Nuclear Medicine and Molecular Imaging*.

[60] Graf R, Plotkin M, Steffen IG (2012). Magnetic resonance imaging, computed tomography, and ^68^Ga-DOTATOC positron emission tomography for imaging skull base meningiomas with infracranial extension treated with stereotactic radiotherapy—a case series. *Head and Face Medicine*.

[61] Henze M, Schuhmacher J, Hipp P (2001). PET imaging of somatostatin receptors using [^68^GA]DOTA-D-Phe1-Tyr3-Octreotide: first results in patients with meningiomas. *Journal of Nuclear Medicine*.

[62] Henze M, Dimitrakopoulou-Strauss A, Milker-Zabel S (2005). Characterization of ^68^Ga-DOTA-D-Phe1-Tyr 3-octreotide kinetics in patients with meningiomas. *Journal of Nuclear Medicine*.

[63] Hänscheid H, Sweeney RA, Flentje M (2012). PET SUV correlates with radionuclide uptake in peptide receptor therapy in meningioma. *European Journal of Nuclear Medicine and Molecular Imaging*.

[64] Conry BG, Papathanasiou ND, Prakash V (2010). Comparison of ^68^Ga-DOTATATE and ^18^F- fluorodeoxyglucose PET/CT in the detection of recurrent medullary thyroid carcinoma. *European Journal of Nuclear Medicine and Molecular Imaging*.

[65] Treglia G, Castaldi P, Villani MF (2012). Comparison of ^18^F-DOPA, ^18^F-FDG and ^68^Ga-somatostatin analogue PET/CT in patients with recurrent medullary thyroid carcinoma. *European Journal of Nuclear Medicine and Molecular Imaging*.

[66] Miederer M, Seidl S, Buck A (2009). Correlation of immunohistopathological expression of somatostatin receptor 2 with standardised uptake values in ^68^Ga-DOTATOC PET/CT. *European Journal of Nuclear Medicine and Molecular Imaging*.

[67] Koukouraki S, Strauss LG, Georgoulias V (2006). Evaluation of the pharmacokinetics of ^68^Ga-DOTATOC in patients with metastatic neuroendocrine tumours scheduled for 90Y-DOTATOC therapy. *European Journal of Nuclear Medicine and Molecular Imaging*.

[68] Middendorp M, Selkinski I, Happel C, Kranert WT, Grünwald F (2010). Comparison of positron emission tomography with [^18^F]FDG and [^68^Ga]DOTATOC in recurrent differentiated thyroid cancer: preliminary data. *Quarterly Journal of Nuclear Medicine and Molecular Imaging*.

[69] Gabriel M, Andergassen U, Putzer D (2010). Individualized peptide-related-radionuclide-therapy concept using different radiolabelled somatostatin analogs in advanced cancer patients. *Quarterly Journal of Nuclear Medicine and Molecular Imaging*.

[70] Versari A, Sollini M, Frasoldati A Differentiated thyroid cancer: a new perspective with radiolabeled somatostatin analogues for imaging and treatment of patients.

[71] Vasamiliette J, Hohenberger P, Schoenberg S (2009). Treatment monitoring with ^18^F-FDG PET in metastatic thymoma after 90Y-Dotatoc and selective internal radiation treatment (SIRT). *Hellenic Journal of Nuclear Medicine*.

[72] Dutta R, Kumar A, Julka PK (2010). Thymic neuroendocrine tumour (carcinoid): clinicopathological features of four patients with different presentation. *Interactive Cardiovascular and Thoracic Surgery*.

[73] Froio A, Sollini M, Fraternali A (2013). Thymic neoplasms evaluation: role of ^68^Ga-peptide and [18F]FDG PET/CT. *Journal of Nuclear Medicine*.

[74] Schneider C, Schlaak M, Bludau M, Markiefka B, Schmidt MC (2012). ^68^Ga-DOTATATE-PET/CT positive metastatic lymph node in a 69-year-old woman with Merkel cell carcinoma. *Clinical Nuclear Medicine*.

[75] Schmidt MC, Uhrhan K, Markiefka B (2012). ^68^Ga-DotaTATE PET-CT followed by Peptide Receptor Radiotherapy in combination with capecitabine in two patients with Merkel Cell Carcinoma. *International Journal of Clinical and Experimental Medicine*.

[76] Salavati A, Prasad V, Schneider C-P, Herbst R, Baum RP (2012). Peptide receptor radionuclide therapy of Merkel cell carcinoma using 177lutetium-labeled somatostatin analogs in combination with radiosensitizing chemotherapy: a potential novel treatment based on molecular pathology. *Annals of Nuclear Medicine*.

[77] Epstude M, Tornquist K, Riklin C (2013). Comparison of, (18)F-FDG PET/CT and (68)Ga-DOTATATE PET/CT imaging in metastasized merkel cell carcinoma. *Clinical Nuclear Medicine*.

[78] Elgeti F, Amthauer H, Denecke T (2008). Incidental detection of breast cancer by ^68^Ga-DOTATOC-PET/CT in women suffering from neuroendocrine tumours. *NuklearMedizin*.

[79] Casini Raggi C, Calabrò A, Renzi D (2002). Quantitative evaluation of somatostatin receptor subtype 2 expression in sporadic colorectal tumor and in the corresponding normal mucosa. *Clinical Cancer Research*.

[80] Haug A, Cindea-Drimus R, Auernhammer C, Schmidt G, Bartenstein P, Hacker M (2012). ^68^Ga-DOTATATE PET/CT in the diagnosis of recurrent neuroendocrine tumors. *Journal of Nuclear Medicine*.

[81] Desai K, Watkins J, Woodward N (2011). Use of molecular imaging to differentiate liver metastasis of colorectal cancer metastasis from neuroendocrine tumor origin. *Journal of Clinical Gastroenterology*.

[82] Brogsitter C, Zöphel K, Wunderlich G, Kämmerer E, Stein A, Kotzerke J (2013). Comparison between F-18 fluorodeoxyglucose and Ga-68 DOTATOC in metastasized melanoma. *Nuclear Medicine Communications*.

[83] Souvatzoglou M, Maurer T, Treiber U, Weirich G, Krause BJ, Essler M (2009). ^68^Ga-DOTATOC-PET/CT detects neuroendocrine differentiation of prostate cancer metastases. *Nuklearmedizin*.

[84] Luboldt W, Zöphel K, Wunderlich G, Abramyuk A, Luboldt H-J, Kotzerke J (2010). Visualization of somatostatin receptors in prostate cancer and its bone metastases with Ga-68-DOTATOC PET/CT. *Molecular Imaging and Biology*.

[85] Alonso O, Gambini JP, Lago G, Gaudiano J, Quagliata A, Engler H (2011). In vivo visualization of somatostatin receptor expression with Ga-68-DOTA-TATE PET/CT in advanced metastatic prostate cancer. *Clinical Nuclear Medicine*.

[86] Von Falck C, Rodt T, Rosenthal H (2008). ^68^Ga-DOTANOC PET/CT for the detection of a mesenchymal tumor causing oncogenic osteomalacia. *European Journal of Nuclear Medicine and Molecular Imaging*.

[87] Woff E, Garcia C, Tant L (2010). Imaging of tumour-induced osteomalacia using a gallium-68 labelled somatostatin analogue. *BMJ Case Reports*.

[88] Clifton-Bligh RJ, Hofman MS, Duncan E (2013). Improving diagnosis of tumor-induced osteomalacia with Gallium-68 DOTATATE PET/CT. *The Journal of Clinical Endocrinology & Metabolism*.

[89] Mawrin C, Schulz S, Pauli SU (2004). Differential expression of sst1, sst2A, and sst3 somatostatin receptor proteins in low-grade and high-grade astrocytomas. *Journal of Neuropathology and Experimental Neurology*.

[90] Kumar U, Grigorakis SI, Watt HL (2005). Somatostatin receptors in primary human breast cancer: quantitative analysis of mRNA for subtypes 1–5 and correlation with receptor protein expression and tumor pathology. *Breast Cancer Research and Treatment*.

[91] Qiu C-Z, Zhu S-Z, Wu Y-Y, Wang C, Huang Z-X, Qiu J-L (2006). Relationship between somatostatin receptor subtype expression and clinicopathology, Ki-67, Bcl-2 and p53 in colorectal cancer. *World Journal of Gastroenterology*.

[92] Pazaitou-Panayiotou K, Tiensuu Janson E, Koletsa T (2012). Somatostatin receptor expression in non-medullary thyroid carcinomas. *Hormones (Athens)*.

[93] Szepeshazi K, Schally AV, Nagy A, Wagner BW, Bajo AM, Halmos G (2003). Preclinical evaluation of therapeutic effects of targeted cytotoxic analogs of somatostatin and bombesin on human gastric carcinomas. *Cancer*.

[94] Reubi JC, Waser B, Schaer J-C, Laissue JA (2001). Somatostatin receptor sst1-sst5 expression in normal and neoplastic human tissues using receptor autoradiography with subtype-selective ligands. *European Journal of Nuclear Medicine*.

[95] Palmieri G, Montella L, Aiello C (2007). Somatostatin analogues, a series of tissue transglutaminase inducers, as a new tool for therapy of mesenchimal tumors of the gastrointestinal tract. *Amino Acids*.

[96] Florio T, Montella L, Corsaro A (2003). In vitro and in vivo expression of somatostatin receptors in intermediate and malignant soft tissue tumors. *Anticancer Research*.

[97] Bläker M, Schmitz M, Gocht A (2004). Differential expression of somatostatin receptor subtypes in hepatocellular carcinomas. *Journal of Hepatology*.

[98] Dalm VASH, Hofland LJ, Mooy CM (2004). Somatostatin receptors in malignant lymphomas: targets for radiotherapy?. *Journal of Nuclear Medicine*.

[99] Lum SS, Fletcher WS, O’Dorisio MS, Nance RW, Pommier RF, Caprara M (2001). Distribution and functional significance of somatostatin receptors in malignant melanoma. *World Journal of Surgery*.

[100] Arena S, Barbieri F, Thellung S (2004). Expression of somatostatin receptor mRNA in human meningiomas and their implication in in vitro antiproliferative activity. *Journal of Neuro-Oncology*.

[101] Papotti M, Macri L, Pagani A, Aloi F, Bussolati G (1999). Quantitation of somatostatin receptor type 2 in neuroendocrine (Merkel cell) carcinoma of the skin by competitive RT-PCR. *Endocrine Pathology*.

[102] Zatelli MC, Tagliati F, Taylor JE, Rossi R, Culler MD, Degli Uberti EC (2001). Somatostatin receptor subtypes 2 and 5 differentially affect proliferation in vitro of the human medullary thyroid carcinoma cell line TT. *Journal of Clinical Endocrinology and Metabolism*.

[103] Mato E, Matías-Guiu X, Chico A (1998). Somatostatin and somatostatin receptor subtype gene expression in medullary thyroid carcinoma. *Journal of Clinical Endocrinology and Metabolism*.

[104] Ferone D, Arvigo M, Semino C (2005). Somatostatin and dopamine receptor expression in lung carcinoma cells and effects of chimeric somatostatin-dopamine molecules on cell proliferation. *American Journal of Physiology—Endocrinology and Metabolism*.

[105] Dizeyi N, Konrad L, Bjartell A (2002). Localization and mRNA expression of somatostatin receptor subtypes in human prostatic tissue and prostate cancer cell lines. *Urologic Oncology*.

[106] Kosari F, Munz JMA, Savci-Heijink CD (2008). Identification of prognostic biomarkers for prostate cancer. *Clinical Cancer Research*.

[107] Saveanu A, Jaquet P (2009). Somatostatin-dopamine ligands in the treatment of pituitary adenomas. *Reviews in Endocrine and Metabolic Disorders*.

[108] Tateno T, Kato M, Tani Y, Oyama K, Yamada S, Hirata Y (2009). Differential expression of somatostatin and dopamine receptor subtype genes in adrenocorticotropin (ACTH)-secreting pituitary tumors and silent corticotroph adenomas. *Endocrine Journal*.

[109] Fusco A, Gunz G, Jaquet P (2008). Somatostatinergic ligands in dopamine-sensitive and -resistant prolactinomas. *European Journal of Endocrinology*.

[110] Yoshihara A, Isozaki O, Hizuka N (2007). Expression of type 5 somatostatin receptor in TSH-secreting pituitary adenomas: a possible marker for predicting longterm response to octreotide therapy. *Endocrine Journal*.

[111] Saveanu A, Morange-Ramos I, Gunz G, Dufour H, Enjalbert A, Jaquet P (2001). A luteinizing hormone-, alpha-subunit- and prolactin-secreting pituitary adenoma responsive to somatostatin analogs: in vivo and in vitro studies. *European Journal of Endocrinology*.

[112] Florio T, Barbieri F, Spaziante R (2008). Efficacy of a dopamine-somatostatin chimeric molecule, BIM-23A760, in the control of cell growth from primary cultures of human non-functioning pituitary adenomas: A Multi-Center Study. *Endocrine-Related Cancer*.

